# The long-term survival of stage IV gastric cancer patients with conversion therapy

**DOI:** 10.1007/s10120-017-0738-1

**Published:** 2017-06-14

**Authors:** Kazuya Yamaguchi, Kazuhiro Yoshida, Toshiyuki Tanahashi, Takao Takahashi, Nobuhisa Matsuhashi, Yoshihiro Tanaka, Kazuaki Tanabe, Hideki Ohdan

**Affiliations:** 1 Department of Surgical Oncology, Gifu University, 1-1 Yanagido, Gifu, 501-1194 Japan; 2 Division of Frontier Medical Science, Department of Surgery, Graduate School of Biomedical Sciences Hiroshima University, Hiroshima, Japan

**Keywords:** Gastric cancer, Conversion therapy, Adjuvant surgery, Chemotherapy, Metastatic gastric cancer

## Abstract

**Purpose:**

A retrospective study was performed to clarify the role of conversion therapy (surgery with a prospect of R0 resection performed in initially unresectable metastatic cancer that responded to the chemotherapy) in stage IV gastric cancer (GC).

**Patients and methods:**

We treated 259 stage IV GC patients with systemic chemotherapy at Gifu and Hiroshima University Hospitals between 2001–2013. Of these, 84 patients who were subsequently treated by surgery were classified into four categories according to our previously published classification of stage IV GC, and short- and long-term outcomes were analyzed.

**Results:**

Surgery was performed in 84 patients, of which 7 were performed following the neoadjuvant chemotherapy, whereas the other 77 that excluded neoadjuvant chemotherapy cases were considered the conversion therapy. The postoperative mortality and morbidity were comparable with those reported clinical trials. The MSTs of the patients with/without surgery for each category were 28.3/5.8 months for category 1, 30.5/11.0 months for category 2, 31.0/18.5 months for category 3 and 24.7/10.0 months for category 4. The MST of the R0 resected patients (41.3 months) was far better than that of the R1–2 resected patients (21.2 months). The MSTs of the patients with R0/R1–2 resection were 56.2/16.3 months for category 2, 33.3/29.6 months for category 3 and 40.7/17.8 months for category 4.

**Conclusion:**

There were long-term survivors who underwent conversion therapy for stage IV GC. Adequate selection of stage IV GC patients for conversion therapy may be an important role for the surgical oncologist in the new era.

## Introduction

According to the GLOBOCAN 2012 database, gastric cancer (GC) is the sixth-ranked cancer in the incidence rate and the fourth in the mortality, and more than 70% of GC cases occur in Asian populations. In Japan, a recent demographic survey showed that GC is the third leading cause of cancer death and the second most common cancer in incidence [1]. Chemotherapy remains the main therapeutic approach for stage IV GC [2], and, as stated in the Japanese treatment guidelines, surgery for this cohort is usually confined to a palliative resection or bypass operation to relieve symptoms [3–5]. Important evidence to support this policy is the REGATTA trial [6], a phase III study in which the investigators attempted to prove the superiority of palliative gastrectomy followed by chemotherapy over chemotherapy alone for stage IV GC with a single incurable factor (metastases to the liver, peritoneal surface or the para-aortic lymph nodes). The surgery-first approach for this cohort was denied as a consequence of that trial.

However, despite recent developments in chemotherapy, median survival time of patients with stage IV GC remains to be around 13–16 months [7–12]. Given that the development of acquired chemo-resistance or cumulative adverse events will eventually render continuation of chemotherapy inadequate, surgery has been offered at some time point during the entire treatment as a part of a multimodality treatment strategy for selected patients who responded well to the chemotherapy [13–20]. To date, the feasibility and survival benefit of gastrectomy with or without metastasectomy for such patients, defined as conversion surgery [13], has not been addressed in prospective clinical trials.

Stage IV GC is a mixed population consisting of patients with various extents of tumor load that were disseminated through diverse metastatic routes. Moreover, some stage IV patients were treated by the neoadjuvant chemotherapy that was not synonymous with the concept of palliative chemotherapy followed by conversion surgery. We therefore proposed a novel classification of stage IV GC into four categories to facilitate comparisons within each category of patients and to discuss the optimal treatment strategies [13].

In the present study, to clarify the feasibility and efficacy of the conversion surgery, stage IV GC patients were classified into the four categories and were analyzed from the viewpoint of the short- and long-term outcomes following surgery.

## Patients and methods

### Patients

We treated 283 patients with stage IV gastric or esophago-gastric junction adenocarcinoma (GC) at Gifu and Hiroshima University Hospitals from 2001 to 2013. Of these, 8 patients were treated with the best supportive care and 16 were treated primarily with palliative gastrectomy; the remaining 259 patients who were treated with chemotherapy formed the basis of the current retrospective study.

The clinical stage classification and pathologic diagnosis of resected specimens were carried out in accordance with the Japanese Classification of Gastric Carcinoma, 14th Edition [2]. All procedures were conducted in accordance with the ethical standards of the respective committees on human experimentation (institutional and national) and with the Helsinki Declaration of 1964 and later versions. The study was approved by the Institutional Review Board of Gifu and Hiroshima Universities.

## Treatment regimens and the response evaluation

The first-line chemotherapies delivered were S-1/cisplatin (SP) [7], S-1/docetaxel (DS) [8, 9], capecitabine plus cisplatin (XP) with or without trastuzumab [11], S-1 plus irinotecan (IRI-S) [21] and S-1 plus docetaxel, cisplatin (DCS) [12] and cisplatin/paclitaxel [22], with the doses and treatment schedules as described previously.

Responses were classified according to the Response Evaluation Criteria in Solid Tumors (RECIST) guidelines [23] and the guidelines of the Japanese Gastric Cancer Association [2]. Tumor size was measured by computed tomography (CT) with a 5-mm slice thickness for all measurable lesions to assess responses every 4–6 weeks as in general clinical practice. The adverse events of chemotherapy were assessed according to the National Cancer Institute-Common Terminology Criteria for Adverse Events (NCI-CTCAE version 4.0) [24].

### New categories of classification for stage IV GC

Stage IV GC patients were classified into four categories according to our previously reported classification [13]. In brief, the patients were primarily classified based on the absence (categories 1 and 2) or presence (categories 3 and 4) of macroscopic peritoneal dissemination. Category 1 included patients with solitary liver metastasis of <5 cm, cytology positive status or metastases to the para-aortic lymph nodes in the region between the celiac axis and the inferior mesenteric artery (no. 16a2 and/or 16b1). This category can be regarded as technically resectable metastasis. Category 2 included patients without apparent peritoneal disease who have metastatic lesions that are regarded as oncologically and technically unresectable. Patients with liver metastases with tumor size >5 cm, more than two nodules, or those located close to the hepatic and/or portal veins were included. As for the lymph node metastasis, patients with metastasis of para-aortic lymph nodes extending to no. 16a1 and 16b2 or with metastases to other distant lymph nodes such as the mediastinum, supraclavicular and axillar metastasis were included. Patients with metastasis to other distant organs with the exception of peritoneal metastases were also included in this category. Category 3 included patients with macroscopic peritoneal dissemination, which is often found at the time of routine screening before operation or at the time of operation or staging laparoscopy, but without other distant metastases. Category 4 included patients with macroscopically peritoneal dissemination and other organ metastases.

### Surgical intervention and indication for conversion therapy

Some patients in category 1 were considered eligible for neoadjuvant chemotherapy according to the recent neoadjuvant trials conducted in Japan and underwent gastrectomy plus metastasectomy or para-aortic lymph node dissection after chemotherapy. For other categories, the patients were initially regarded as unresectable, but some patients responded so well to the chemotherapy that gastrectomy and/or metastasectomy could be considered. The patients were offered surgery, referred to here as conversion surgery, when preoperative imaging studies and staging laparoscopy (in case peritoneal metastases had been detected) after several courses of chemotherapy suggested that R0 resection might be possible. Conversion surgery could therefore be defined as surgical treatment aiming at R0 resection for originally unresectable or marginally resectable tumors after exceptionally good response to the chemotherapy. Only gastrectomy with lymph node dissection was performed for stage IV GC patients with distant metastasis where CR was observed as a result of chemotherapy. This was often the case for patients who belong to categories 3 and 4 where peritoneal disease had initially been diagnosed.

The postoperative complications were evaluated according to the Clavien-Dindo classification [25]. Postoperative adjuvant chemotherapy was basically performed with S-1 monotherapy or S-1 and another drug until relapse, depending on the curability of the surgery.

### Follow-up data and statistical analyses

Medical records were retrospectively reviewed to determine the clinicopathological features and surgical findings based on the Japanese Classification of Gastric Carcinoma 14th Edition [2]. The overall survival (OS) period was defined as the time from the initiation of chemotherapy to death or the day of the last follow-up. Regular CT scans were performed every 3 months with other radiological modalities or a gastrointestinal fiberscope.

The OS was calculated using the Kaplan-Meier method, and the survival was compared using the log-rank test. The OS was calculated from the date of chemotherapy initiation to the date of all-cause death or the latest follow-up. The median follow-up period was 22.9 months (range 0.6–139 months) for all patients and 28.5 months (range 7.5–139 months) for those who underwent surgery. All statistical analyses were performed using the SPSS 11.5J software package (SPSS Japan Inc. Tokyo, Japan). A *p* value <0.05 (two-sided) was defined as statistically significant.

## Results

### Patient demographics and efficacy of chemotherapy

**Fig. 1:**
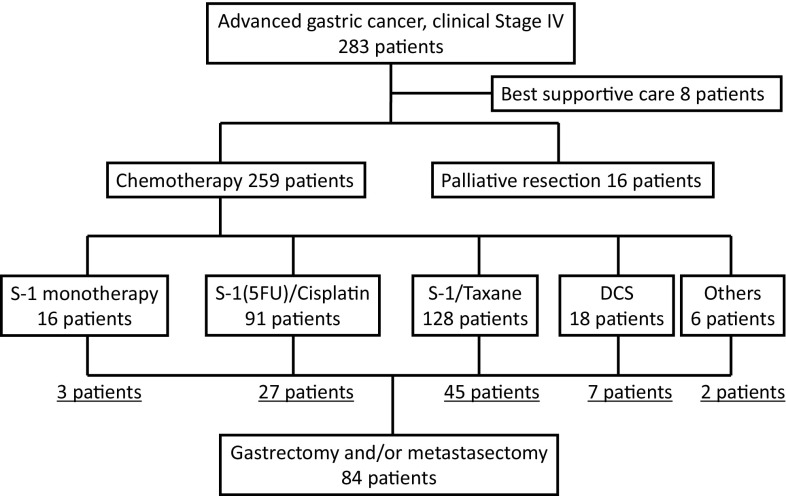
Consort diagram. A total of 259 patients were enrolled in the present cohort study, and 84 received conversion therapy. #1: DCS, docetaxel/cisplatin/S-1 therapy. #2: Others, XP(+Tmab) 4 patients, S-1/CPT-11 1 patient, cisplatin/paclitaxel 1 patient

Figure 1 shows the consort diagram of the patients enrolled in this study. All of the patients were diagnosed with stage IV GC according to the Japanese classifications and treatment guidelines for GC. Sixteen patients underwent palliative resection, and eight patients received best supportive care. The remaining 259 patients who were treated with chemotherapy were analyzed in the current study. Regarding the regimens, 128 patients were treated with S-1/docetaxel, 91 with S-1/cisplatin, 18 with DCS and 22 with other regimens (S-1 monotherapy, XP ± trastuzumab, IRI-S, cisplatin/paclitaxel). Surgery fulfilled our definition of conversion surgery in 77 patients, while the treatment strategy of the 7 other patients was considered as neoadjuvant chemotherapy followed by surgery (45 after S-1/docetaxel, 27 after S-1/cisplatin, 7 after DCS and 5 after other regimens).

The demographics of all patients treated with chemotherapy, those who underwent conversion therapy and those who were not treated by surgery are listed in Table 1, and the treatment efficacy is described in Table 2. The overall response rate (ORR) of the patients was 36.3%, and the disease control rate (DCR) was 86.1%. Concerning the 84 patients who received surgery, the ORR was 58.3% [complete response (CR) 3.6% and partial response (PR) 54.7%], stable disease (SD) was 39.3%, and progressive disease (PD) was 2.4%. Thus, the DCR was 97.6%.

**Table 1:** Clinicopathological findings

	Chemotherapy *n* = 259 (all patients)	Surgery *n* = 84	Non-resected *n* = 175 (chemotherapy alone)	*p* value
Age (years)				
Mean	63.4	61.7	64.1	
Range	21–89	21–78	25–89	0.1225
Gender				
Male	177	56	121	
Female	82	28	54	0.6898
Lauren type				
Intestinal	97	32	65	
Diffuse	151	49	102	
Mixed	11	3	8	0.6641
Depth of tumor invasion (T)				
T1	0	0	0	
T2	1	1	0	
T3	25	11	14	
T4	225	72	153	0.9111
TX	8	0	8	
Lymph node metastasis (N)				
N0	16	7	9	
N1	55	23	32	
N2	75	26	49	
N3	105	28	77	0.5519
NX	8	0	8	
Peritoneal metastasis (P)				
P0	144	49	95	
P1	115	35	80	0.5519
Hepatic metastasis (H)				
H0	166	64	102	
H1	93	20	73	0.0182
Distant metastasis (M)				
M0	100	38	62	
M1	159	46	113	0.2014

**Table 2:** Response to chemotherapy

	Chemotherapy *n* = 259 (%) (all patients)	Surgery *n* = 84 (%)	Non-resected *n* = 175 (%) (chemotherapy alone)
CR	3 (1.2)	3 (3.6)	0 (0)
PR	91 (35.1)	46 (54.7)	45 (25.7)
SD	129 (49.8)	33 (39.3)	96 (54.9)
PD	36 (13.9)	2 (2.4)	34 (19.4)
DCR (%)	86.1	97.6	80.6
ORR (%)	36.3	58.3	25.7

Mann-Whitney *U* test *p* < 0.0001 (comparison of response between surgery and non-resected)

*CR* complete response, *PR* partial response, *SD* stable disease, *PD* progression disease, *DCR* disease control rate, *ORR* overall response rate

### Surgical complications in patients with conversion therapy

Table 3 shows the tumor burdens and the backgrounds of the 84 patients who received operations. The metastatic sites were liver metastasis in 20 patients, peritoneal dissemination in 35, distant lymph nodes in 37 and other organs in 14. The other metastatic sites were positive lavage cytology without macroscopic evidence of peritoneal metastasis in ten patients, metastasis to the lung, bone and ovary in one patient each, and tumor embolism in the portal vein in one patient. The median number of distant metastatic sites was one. Distal gastrectomy was performed in 15 patients and total gastrectomy in 69 patients. The median (range) operation time was 254 min (range 133–854), and the median bleeding amount was 360 ml (range 50–3150). An R0 operation was performed in 43 patients (51.2%). Metastasectomy of one organ with a primary lesion was performed in 6 patients and metastasectomies of two or more organs were performed in 9 patients, while only gastrectomy plus regional lymphadenectomy was performed in the remaining 69 patients.

**Table 3:** Tumor burden of baseline and operative findings of patients with surgery

	Surgery *n* = 84
Tumor burden of baseline	
Metastatic organ	
Liver	20
Peritoneal dissemination	35
Distant lymph nodes	37
Other organ	14
Number of metastatic organs	1–3 (median 1)
Operation	
Type of gastrectomy	
Distal	15
Total	69
Operation time (min)	133–854 (median 254)
Amount of bleeding (g)	50–3150 (median 360)
Curability	
R0	43
R1–2	41
Other organs resection	
0	69
1	6
≥2	9

Surgery as conversion therapy was safely conducted in the present study without perioperative mortality, with postoperative mortalities as shown in Table 4. Operative complications of all grades amounted to 24 events. One grade 3 and one grade 4 pancreatic fistula were observed after the operation. One grade 3 anastomotic leakage, one grade 3 bowel obstruction, one grade 3 and one grade 4 pulmonary infection after surgery occurred in the present study. These rates are comparable to the postoperative comorbidity rates reported previously in a phase III surgical trial [26].

**Table 4:** Postoperative complications

	Total	Grade 1	Grade 2	Grade 3	Grade 4
Pancreatic fistula	3		1	1	1
Anastomotic leakage	1			1	
Anastomotic bleeding	1		1		
Anastomotic stricture	1		1		
Intra-abdominal abscess	1		1		
Bowel obstruction	6	4	1	1	
Wound infection	4	4			
Pulmonary infection	2			1	1
Pulmonary embolism	1				1
Sepsis	2		2		
Stasis	1		1		
Atrial fibrillation	1		1		

### Survival of the patients with conversion therapy

**Fig. 2:**
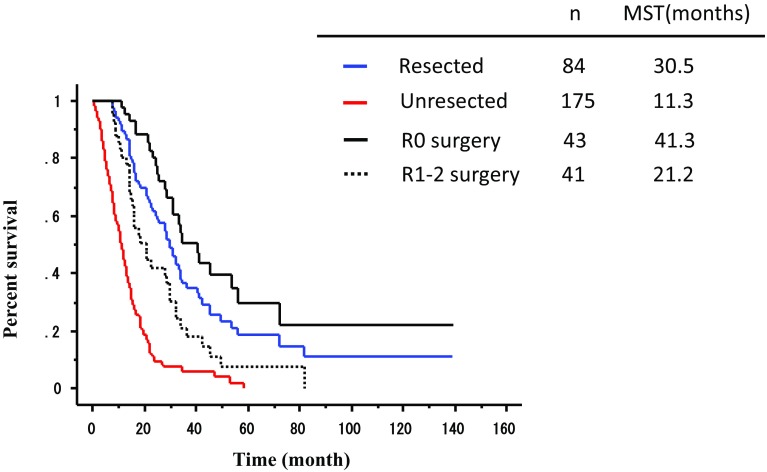
Survival curve of all patients enrolled in the present analysis. Survival curve of the patients who received and did not receive conversion therapy. The MST of the patients with conversion surgery and NAC patients (resected) was 30.5 months, and it was 11.3 months in those without conversion surgery (unresected). The MST of R0 resected patients (R0 surgery) was 41.3 months and that of patients with R1 and R2 resection (R1–2 surgery) was 21.2 months

The MST of all 259 patients in the present study was 15.1 months. The MST of the patients who underwent surgical resection was 30.5 months, as opposed to those who received chemotherapy alone at 11.3 months, which is consistent with the MST reported previously in clinical trials for advanced/metastatic cancer treated with the first-line chemotherapy [9, 10] (Fig. 2). Furthermore, the MST of R0 resected patients at 41.3 months was significantly better than that of patients with R1 and R2 resection (21.2 months), as shown in Fig. 2. As expected, the prognosis of the patients in whom PR was achieved with the initial chemotherapy was far better than in those with SD or PD (data not shown).

.

However, given that stage IV GC is composed of a mixture of hematological, peritoneal and distant lymph node metastases, each cancer should be treated according to their cancer volume and biological characteristics. We therefore proposed a new classification of stage IV GC, as described in the methods. Consequently, the 259 patients were classified as follows: category 1, 9 patients; category 2, 135 patients; category 3, 31 patients; category 4, 84 patients. The MST of the patients in category 1, 2, 3 and 4 were 26.3, 14.8, 22.0 and 12.9 months, respectively.

**Fig. 3:**
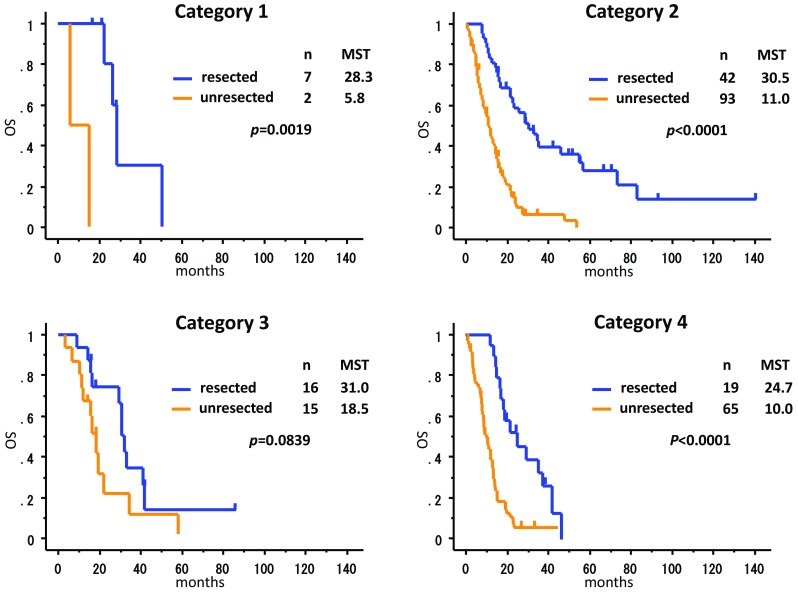
Survival curve of the patients according to the new categories of classification for stage IV GC. The MST of patients with operation (*n* = 7) in category 1 was 28.3 months, while that of patients without surgery (*n* = 2) was 5.8 months. In category 2, the MST with conversion therapy (*n* = 42) was 30.5 months and that of patients without surgery (*n* = 93) was 11.0 months. The MST of category 3 patients with surgery (*n* = 16) was 31.0 months and that of patients without surgery (*n* = 15) was 18.5 months. The MST of category 4 patients with surgery (*n* = 19) was 24.7 months and that of patients without surgery (*n* = 65) was 10.0 months

Interestingly, the MST of category 1 patients treated with the neoadjuvant chemotherapy followed by surgery was 28.3 months, while that of patients without surgery in this category was only 5.8 months (Fig. 3). The MST of category 2 patients treated with conversion surgery was 30.5 months, whereas that of patients without surgery was 11.0 months. The MST of category 3 patients with surgery was 31.0 months and that of patients without surgery was 18.5 months. The MST of category 4 patients with surgery was 24.7 months and that of patients without surgery was 10.0 months. The R0 resection rate was 85.7% (6/7) in category 1, 52.4% (22/42) in category 2, 50.0% (8/16) in category 3 and 36.8% (7/19) in category 4. MST of the patients who underwent R0 resection as opposed to those who underwent R1/R2 resection was 56.2 versus 16.3 months for category 2, 33.3 versus 29.6 months for category 3 and 40.7 versus 17.8 months for category 4. Although the number of patients in each category was too small for statistical analyses, MST of the patients treated by R0 operations in each of the categories was superior compared with that of the patients treated by R1/R2 operation.

## Discussion

In the present study, planned resection after neoadjuvant chemotherapy was performed in 7 patients and conversion surgery was performed in another 77 patients among 259 stage IV GC patients who were initially treated by chemotherapy. Although only 43 patients (51.2% of the patients who received surgery) underwent R0 resection, MST of these patients at 41.3 months was much longer than that reported from the first-line chemotherapy trials [9–11].

In the current series, the outcome of patients in category 1 was not necessarily favorable when compared with patients in other categories with greater tumor volume, although this was the only population where neoadjuvant chemotherapy followed by complete resection of the metastatic lesions could be expected. The number of patients in this category was the smallest of all four categories, indicating that stage IV GC can rarely be in a favorable tumor status so as to be indicated for the neoadjuvant strategy. Whether the neoadjuvant strategy is adequate for this population or the patients for radical surgery should be more carefully selected through longer duration of chemotherapy as in other categories (the conversion strategy) could be another issue for debate. One could conclude at least that stage IV GC remains a formidable opponent even when the tumor volume seems to be smaller. Lastly, whether to include cytology positive cases into this category or to the category 3 remains controversial, given that surgery that could only be justified when the cytology turns negative would be considered as more suitable for conversion strategy rather than the neoadjuvant strategy.

With the R0 resection rate of 52.4% among those sent for surgery, the decision of whether the R0 operation is possible remains to be a difficult task for category 2 patients. All efforts to detect metastatic lesions should be made with the modern diagnostic tools such as CT, MRI and PET-CT before venturing on to surgical intervention for the patients in this category. Metastasectomy along with resection of the primary might be feasible for this population provided the metastases have responded well to the chemotherapy, preferably with a small residual tumor volume left for a relatively easy resection. In cases of para-aortic lymph node metastasis extending to the nos. 16a1 and No. 16b2 regions, complete para-aortic lymphadenectomy might be ideal after successful chemotherapeutic treatment. However, this recommendation depends on the patients’ condition, including their nutritional status, age and other comorbidities. In more vulnerable patients, complete lymph node dissection could be replaced by sampling in which only the swollen nodes in the paraaortic region are resected [27–31].

Peritoneal dissemination is usually considered oncologically unresectable, and patients with this pattern of metastasis are classified as categories 3 and 4 in our classification. However, among the category 3 patients, not only was the survival of those who received conversion surgery prolonged at 31.0 months, but even the outcome of those who failed to undergo surgery (18.5 months) was better than what we had expected. Apart from several biases that could have occurred because of the retrospective nature of this study, the favorable outcome can be understood based on the results of a subset analysis of the previous phase III START trial [9], wherein the MST of the patients with non-measurable lesions was around 17 months. This is further supported by the similar findings in the SPIRITS trial [7]. Peritoneal disease with minimal or microscopic deposits detectable only through staging laparoscopy or cytologic examination may be more vulnerable to chemotherapy when compared with overt peritoneal carcinomatosis. Although the definitive indication of total peritonectomy has not yet been clarified as in the case of other peritoneal surface malignancies, gastrectomy may be feasible when the peritoneal deposits disappeared completely or when negative cytology is confirmed based on negative findings by CT or staging laparoscopy. In contrast, patients with other organ involvement in addition to peritoneal disease (classified as category 4) understandably had fewer chances for surgical intervention. Recent reports on the efficacy of intraperitoneal chemotherapy and subsequent surgery [32] suggest that the outcome of category 3 patients could in the future approach that of category 2 patients in which the strength of conversion surgery was most prominently observed in this study.

The present study has several limitations. First, this is a retrospective study with a small sample size, especially in category 1, to explore the role of surgical strategy. Second, due to the heterogeneity in the biology of stage IV GC, the long period of patient accrual and lack of a fixed study protocol, there were inconsistencies regarding the chemotherapeutic regimen, extent of surgical resection and timing of surgery, most of which had been decided on a case-by-case basis. Some patients even proceeded to surgery for various reasons despite the disease progression in the measurable lesion. Third, the indication for and compliance with the postoperative chemotherapy varied. Lastly, the clinical significance of the proposed classification for stage IV GC had not been verified. Details such as the inclusion of cytology-positive status into category 1 should in the future be debated more openly, together with the difference between the conversion therapy and the neoadjuvant strategy, if category 1 is to be distinct from other categories in terms of treatment strategy and clinical outcome.

In conclusion, there were long-term survivors among patients who underwent conversion surgery for stage IV GC. Adequate selection of stage IV GC patients for conversion therapy may be an important role for the surgical oncologist in the new era. We are now conducting a large-scale retrospective cohort study in Asian countries based on the Federation of Asian Clinical Oncology (FACO), which includes the Japanese Society of Clinical Oncology (JSCO), Korean Association of Clinical Oncology (KACO) and Chinese Society of Clinical Oncology (CSCO), supported by the Japanese Gastric Cancer Association (JGCA), Korean Gastric Cancer Association (KGCA) and Gastric Cancer Association of the Chinese Anti-cancer Association. Analysis of this data, in which our classification of stage IV GC could be of help, may deepen our understanding of the role of conversion surgery for stage IV GC and pave the way for conducting relevant clinical trials to further explore this strategy.
